# Diarrhoeagenic *E. coli* occurrence and antimicrobial resistance of Extended Spectrum Beta-Lactamases isolated from diarrhoea patients attending health facilities in Accra, Ghana

**DOI:** 10.1371/journal.pone.0268991

**Published:** 2022-05-26

**Authors:** Helena Dela, Beverly Egyir, Ayodele O. Majekodunmi, Eric Behene, Clara Yeboah, Dominic Ackah, Richard N. A. Bongo, Bassirou Bonfoh, Jakob Zinsstag, Langbong Bimi, Kennedy Kwasi Addo

**Affiliations:** 1 Department of Bacteriology, Noguchi Memorial Institute for Medical Research (NMIMR), University of Ghana, Legon, Accra, Ghana; 2 Department of Animal Biology and Conservation Science (DABCS), University of Ghana, Legon, Accra, Ghana; 3 Institut de Recherche en Elevage pour le Développement (IRED), N’djamena, Chad; 4 Centre Suisse de Recherches Scientifiques en Côte d’Ivoire (CSRS), Abidjan, Côte d’Ivoire; 5 Department of Epidemiology and Public Health (EPH), Swiss TPH, Basel Switzerland; Government College University Faisalabad, PAKISTAN

## Abstract

**Introduction:**

Diarrhoea accounts for high morbidity and mortality in children and adults worldwide. Extended Spectrum Beta-Lactamase-Producing Enterobacteriaceae (ESBL-PE) and Diarrhoeagenic *Escherichia coli* (DEC) contribute to prolonged hospitalization because of their resistance and virulence properties aiding in the spread of diarrhoeal disease and delayed treatment.

**Aim:**

To determine DEC and the antimicrobial resistance of ESBL-PE isolated among diarrhoea patients attending two health facilities in Ghana.

**Methods:**

Stool samples were collected from 122 diarrhoeal patients who attended Maamobi General Hospital and Kaneshie Polyclinic between January 2019 and March 2020. Identification of bacteria was performed by using the Matrix-assisted laser desorption ionization-time of flight mass spectrometry (MALDI-TOF MS). Using disk diffusion, antimicrobial susceptibility testing (AST) was conducted and interpreted according to the 2018 CLSI guidelines. Detection of ESBL and DEC genes was performed using Polymerase chain reaction (PCR).

**Results:**

A total of 80.3% (98/122) Enterobacteriaceae was recovered from the patients in the study with an overall ESBL occurrence of 20.4% (20/98), predominantly among *E*. *coli* showed 13.2% (10/76), *Klebsiella pneumoniae*,35.7%(5/14) and *Proteus mirabilis*, 57.1%(4/7). Among the ESBL genes detected, *bla*_*TEM*_ (n = 14) was common, followed by *bla*_*CTX-M*_ (n = 13) and *bla*_*SHV*_ (n = 4). Thirty-four *E*. *coli* isolates possessed the heat labile (Lt) gene of Enterotoxigenic *E*. *coli* (ETEC).

**Conclusion:**

Our findings confirm the existence of DEC and the antimicrobial resistance patterns of ESBL-PE among stool isolates, limiting the options of commonly used drugs for diarrhoeal treatment in Ghana. Routine laboratory testing in health care facilities and strengthened surveillance systems among hospital networks are encouraged for a better understanding of their epidemiology and clinical implications.

## Introduction

Diarrhoea caused by bacterial food pathogens is one of the major reasons why people seek medical attention on a daily basis worldwide [1]. Various food poisonings encountered by patients in Ghana and various parts of the world, have been attributed to bacterial foodborne pathogens such as *Salmonella* spp., *Shigella* spp., *E*. *coli* [2–4]. Other studies have investigated various diarrhoeagenic *E*. *coli* (DEC) and various strains isolated in stool in Ghana and their antimicrobial properties and relationship [5–7]. Diarrhoeagenic *E*. *coli*, like Enterotoxigenic *E*. *coli* (ETEC) has been documented to be the leading cause of traveler’s diarrhoea and acute infant diarrhoea all over the world, commonly resulting in abdominal cramping, watery diarrhoea and fever/ nausea during severe occurrences [8–10]. Acute infections are normally self-limiting within 3–7 days and managed or treated with rest and oral rehydration therapy [11, 12]. Treatment failure with this initial regimen is normally augmented with antibiotic administration which has over the years gradually contributed to the emergence of antimicrobial resistance [13, 14].

Since many patients with enteric diseases are treated with antibiotics prior to AST or in the absence antibiograms, it is important to know the performance of these commonly used antibiotics among the major enteric pathogens [14].

Antimicrobial resistant foodborne pathogens among stool isolates are commonly observed globally as well in Ghana [2, 4, 5, 14–16]. Due to the gradual mutations of some bacteria in their B-lactamases, resistance to beta-lactam antibiotics is currently on the rise resulting in treatment difficulties among Gram-negative bacterial associated infections globally [17]. There are a few reported cases of Extended Spectrum Beta-Lactamase producing Enterobacteriaceae (ESBL-PE) that have been recovered from stool [3, 14, 18–22].

ESBLs can hydrolyze third generation cephalosporins and aztreonam despite their inhibition by clavulanic acid. Based on the functional enzymatic properties of their substrate and inhibitor profiles, over 200 of ESBL genetic variants have been characterized worldwide [23]. Some of the enzymes includes TEM, SHV, CTX-M, OXA-type and their associated mutants resulting in the numerous variants which are individually exhibit resistance to specific antibiotic types [24].

High prevalence of ESBL among stool isolates have been reported in Africa with high numbers of *bla*_*CTX-M-15*_, followed by *bla*_*SHV*_ and *bla*_*CTX-M-14*_ ESBL genes [25]. The spread of ESBL-PE has been known to complicate the treatment regimen and prolong hospitalization and among diarrhoea patients, thus having adverse effect on the socio-economic status and public health [3, 14]. Patients with ESBL-PE and ETEC genes may exhibit treatment failures due to unresponsiveness to first-line antibiotic administration [8].

Using MALDITOF-MS, AST and PCR, this study sought to investigate the occurrence of ESBL-PE and DEC among Enterobacteriaceae recovered from patients with diarrhoea.

## Methods

### Study area

The study was conducted at Maamobi General Hospital and Kaneshie polyclinic in Accra, Ghana. The sites were selected based on history/records of foodborne outbreaks [26].

### Study design

This cross-sectional study was conducted between January 2019 and March 2020. After obtaining an informed consent, patient demographic information such as age, gender, and date of onset of symptoms were captured using a structured questionnaire. Stool samples (loose or liquid) were collected from patients who came to the hospitals with diarhoea or history of diarhoea within the last 24 hours. The samples were collected using receptacles containing Cary-Blair transport medium. Unique study identification numbers were assigned to specimens and transported at 4˚C to the Noguchi Memorial Institute for Medical Research (NMIMR) for further analysis.

### Bacterial isolation, identification, and enumeration

The samples were directly streaked on MacConkey agar (Oxoid, UK) and incubated for 18-24h at 37 ˚C. Identification of bacteria was done using colonial morphology, Gram staining, catalase test, oxidase test and the MALDI-TOF MS System (Bruker, Billerica, MA, USA). Overnight cultures were spotted on the target plate of the MALDI-TOF MS after which 1 μl of formic acid was added and allowed to air dry for 15 min. A matrix preparation (α-cyano-4-hydorxy-cinnamic acid and trifluoroacetic acid)of one (1) μl was added to each sample and allowed to dry for another 15 minutes and placed in the MALDI-TOF MS System. Species of bacteria were confirmed by the ionization peaks that were generated and matched against the integrated reference library of the MALDI-TOF MS equipment.

### Antimicrobial susceptibility testing

Antimicrobial susceptibility testing (AST) was carried out using Kirby Bauer’s Disc Diffusion method and interpreted according to the Clinical and Laboratory Standard Institute (CLSI) guidelines (2018).

The following antibiotics for the Enterobacteriaceae were tested: Ceftriaxone (30μg), Sulfamethoxazole-trimethoprim (25μg), Piperacillin-tazobactam (110 μg), Ticarcillin-clavulanate (85μg), Tetracycline (30μg), Amikacin (30μg), Gentamicin (10μg), Ciprofloxacin (5μg), Meropenem (10μg), Azithromycin (15μg), Chloramphenicol (30μg), Nitrofurantoin (300μg), Nalidixic acid (30μg), Ceftazidime (30μg) and Amoxicillin-clavulanate (30μg) (Oxoid, Basingstoke, Hants, UK). Quality control was ensured by using standard strains (*Klebsiella pneumoniae*, ATCC 700603 and *Escherichia coli*, ATCC 25922) in determining susceptibility or otherwise of stool isolates. The definition of multidrug resistance (MDR) was based on the resistance to three or more classes of antimicrobial agents [27].

### Phenotypic detection of ESBL

The double-disk synergy test was performed using paired discs (MAST, Merseyside, UK). This test was done using both cefotaxime (30μg) and ceftazidime (30μg) alone and in combination with Clavulanic acid (i.e: Ceftazidime 30, Ceftazidime/ Clavulanic acid 30/10 and Cefotaxime 30, Cefotaxime/ Clavulanic acid 30/10) to screen for the presence of ESBLs. After an overnight incubation at 37˚C, a difference of ≥ 5 mm in zone diameter for any of the cephalosporins and those in combination with clavulanic acid, was interpreted as ESBL positive. *Klebsiella pneumoniae*, ATCC 700603 and *Escherichia coli*, ATCC 25922 were used as quality control strains.

### Polymerase chain reaction detection of ESBL and DEC genes

DNA was extracted using the crude method, which involved, collecting 6 colonies of freshly grown pure isolates with a sterile swab into 180 μl of nuclease-free water. The broth was then briefly, vortexed, heated at 100°C for 10mins and centrifuged for 5 min at 14000rpm. The supernatant was transferred to sterile tube and used for both genotypic ESBL and DEC gene testing.

PCR detection of ESBL- genes (Table 1), was done by using 10μM of each primer (Table 1); (*bla*_*CTX-M*_, *bla*_*SHV*_ and *bla*_*TEM)*_ PCR master mix (13μl) (Qiagen, Hilden Germany), PCR grade water (10μl) and 2μl DNA template, in a final volume of 25μl. Using previously described primers and cycling conditions [28], amplification was performed in an Eppendorf Master cycler X50s (Eppendorf, Hamburg, Germany) with cycling conditions as follows; 95°C for 5 minutes, 35 cycles of 95°C for 30 seconds, 60°C for 30 seconds, 72°C for 2 minutes, and a final extension lap at 72°C for 10 minutes. Agarose Gel Electrophoresis was done at 100 volts for 1 hour with a 2% w/v agarose gel concentration. Visualization of the gel was done on a UV platform using Gel Documentation system XR+ (Bio-Rad, USA) using Image lab version 5.0 build 18.

**Table 1:** DEC and ESBL primer sequence details.

Gene	Primer sequence (5’-3’)	Target	Expected product size (bp)
*Stx*_*1*_	CAG TTA ATG TGG TGG CGA AGG	shiga toxin-1 gene of STEC	348
	CAC CAG ACA ATG TAA CCG CTG		
*Stx*_*2*_	ATC CTA TTC CCG GGA GTT TAC G	shiga toxin-2 gene of STEC	584
	GCG TCA TCG TAT ACA CAG GAG C		
*eae*	TCA ATG CAG TTC CGT TAT CAG TT	attaching and effacing lesion of EPEC	482
	GTA AAG TCC GTT ACC CCA ACC TG		
*lt*	GCA CAC GGA GCT CCT CAG TC	Heat labile gene of ETEC	218
	TCC TTC ATC CTT TCA ATG GCT TT		
*stII*	AAA GGA GAG CTT CGT CAC ATT TT	heat stable gene of ETEC	129
	AAT GTC CGT CTT GCG TTA GGA C		
*VirF*	AGC TCA GGC AAT GAA ACT TTG AC	gene responsible for transcription of virulent factors of EIEC	618
	TGG GCT TGA TAT TCC GAT AAG TC		
*ipaH*	CTC GGC ACG TTT TAA TAG TCT GG	invasion plasmid antigen H of EIEC	933
	GTG GAG AGC TGA AGT TTC TCT GC		
*daaE*	GAA CGT TGG TTA ATG TGG GGT AA	fimbrial adhesin gene of DAEC	542
	TAT TCA CCG GTC GGT TAT CAG T		
*aafII*	CAC AGG CAA CTG AAA TAA GTC TGG	aggregative adherence fimbrae- II genes of EAEC	378
	ATT CCC ATG ATG TCA AGC ACT TC		
	*DEC Primer sequences adopted from *Vidal et al*. *(2005)*				
bla_CTX-M_	GAA GGT CAT CAA GAA GGT GCG	cefotaximase (CTX-M) beta-lactamase	560
	GCA TTG CCA CGC TTT TCA TAG		
bla_SHV_	GTC AGC GAA AAA CAC CTT GCC	sulfhydryl (SHV) beta-lactamase	383
	GTC TTA TCG GCG ATA AAC CAG		
bla_TEM_	GAG ACA ATA ACC CTG GTA AAT	temoneira (TEM) beta-lactamase	459
	AGA AGT AAG TTG GCA GCA GTG		
*ESBL Primer sequences adopted from Sharma 2013			

#### Primers for detection of DEC genes

Shiga toxin-producing (*stx*1 and *stx*2), enteropathogenic (*eae*), enterotoxigenic (*stII* and *lt*), enteroinvasive (*virF* and *ipaH*), enteroaggregative (*aafII*), and diffuse adherent (*daaE*) are also shown in Table 1. In a 25μl reaction mixture containing 0.5μl of each primer and deoxynucleoside triphosphates, 5μl MgCl_2_, 0.25μl of Taq DNA polymerase and 3μl of template DNA, PCR was performed based Vidal et al.,2005 with few modifications with the following cycling conditions; 1.5min at 94˚C for denaturation, 1.5min at 60˚C for primer annealing, amplification for 35 cycles, and 1.5 min at 72˚C for strand elongation. Agarose Gel Electrophoresis was done at 100 volts for 1 hour with a 1.5% w/v agarose gel stained with SYBR Safe DNA gel stain (Invitrogen, Thermo Fisher Scientific, USA). Visualization of the gel was done on a UV platform using Gel Documentation system XR+ (Bio-Rad, USA) using Image lab version 5.0 build 18 [29].

### Data quality control and analysis

Standard Operating procedures were adhered to during sample collection, transportation, and laboratory processing. Standard bacteria strains were used to ensure the quality control of AST/ ESBL detection.

### Data analysis

Unique identification numbers were assigned to specimens/ patients. Cleaning and data analysis was performed in STATA, version 13.

Prevalence and antimicrobial resistance profiles in the selected foodborne pathogens in the stool samples was done using frequencies and percentages. Fisher’s exact/ Chi-square tests where necessary was used to determine the association between ESBL carriage, AST, and demographic characteristics.

### Ethical consideration

The study was approved by the Noguchi Memorial Institute for Medical Research (NMIMR) and the Ghana Health Service (GHS) Institutional Review Boards (IRB) with the approval numbers NMIMR-IRB CPN 097/17-18 and GHS-ERC001/08/18 from both institutions, respectively. Patients consented to participating in the study with written consent after detailed explanation in the presence of witnesses who also gave written authorization.

## Results

### Demographic data

A total of 122 patients were enrolled in the study with children and infants forming part of a lower percentage of patients (17.2%) during the study. Females enrolled were more than the males (67.2%, 32.8%). More than half of the patients (52.9%) reported having symptoms of diarrhoea within 24 hours (Table 2).

**Table 2:** The various characteristics of patients versus ESBL associated diarrhoea.

Characteristics	N(%)	ESBL associated diarrhoea	Non ESBL associated diarrhoea	*p-value*
		n(%)	n(%)	
**Gender**				
Male	40(32.8)	9(22.5)	31(11.5)	0.141
Female	82(67.2)	10(12.2)	72(87.8)	
**Age**				
Infant (<5 yrs)	12(9.8)	3(25.0)	9(75.0)	0.015
Children (5-17yrs)	9(7.4)	4(44.4)	5(55.6)	
Adult (≥18 yrs)	101(82.8)	12(11.9)	89(88.1)	
**Site**				
Mamobi Polyclinic	68(55.7)	12(17.7)	56(82.4)	0.617
Kaneshie Polyclinic	54(44.3)	7(13.0)	47(87.0)	
**Duration of symptoms**				
Less than a day	20(16.5)	1(5.0)	19(95.0)	0.337
A day	44(36.4)	10(22.2)	35(77.8)	
Two days	17(14.1)	3(17.7)	14(82.3)	
Three days or more	40(33.1)	5(12.5)	35(87.5)	
**Pathogens**				
*Escherichia coli*	76(62.3)	10(13.2)	66(86.8)	-
*Klebsiella pneumoniae*	14(11.5)	5(35.7)	9(64.3)	-
*Citrobacter freundii*	1(0.8)	1(100)	0(0.0)	-
*Proteus mirabilis*	7(5.7)	4(57.1)	3(42.9)	-

### Pathogen recovery

*E*. *coli* (62.3%; 76/122) was predominant among the pathogens recovered, followed by *K*. *pneumoniae* (11.5%; 14/122), *P*. *mirabilis* (5.7%; 7/122) and *C*. *freundii* (0.8%; 1/122) (Table 2). Although 44.7% (34/76) of *E*. *coli* recovered possessed the Lt gene, 17.6% (6/34) coexisted with ESBL properties. The rest of the ETEC (Lt gene) organisms (82.4%; 28/34), although recovered in the stool of diarrhoeal patients had no coexistence with ESBL properties. None of the demographic characteristics had a significant association with ESBL or Non ESBL diarrhoea except age (p = 0.001) (Table 2).

### Antimicrobial resistance profile

**Fig 1:**
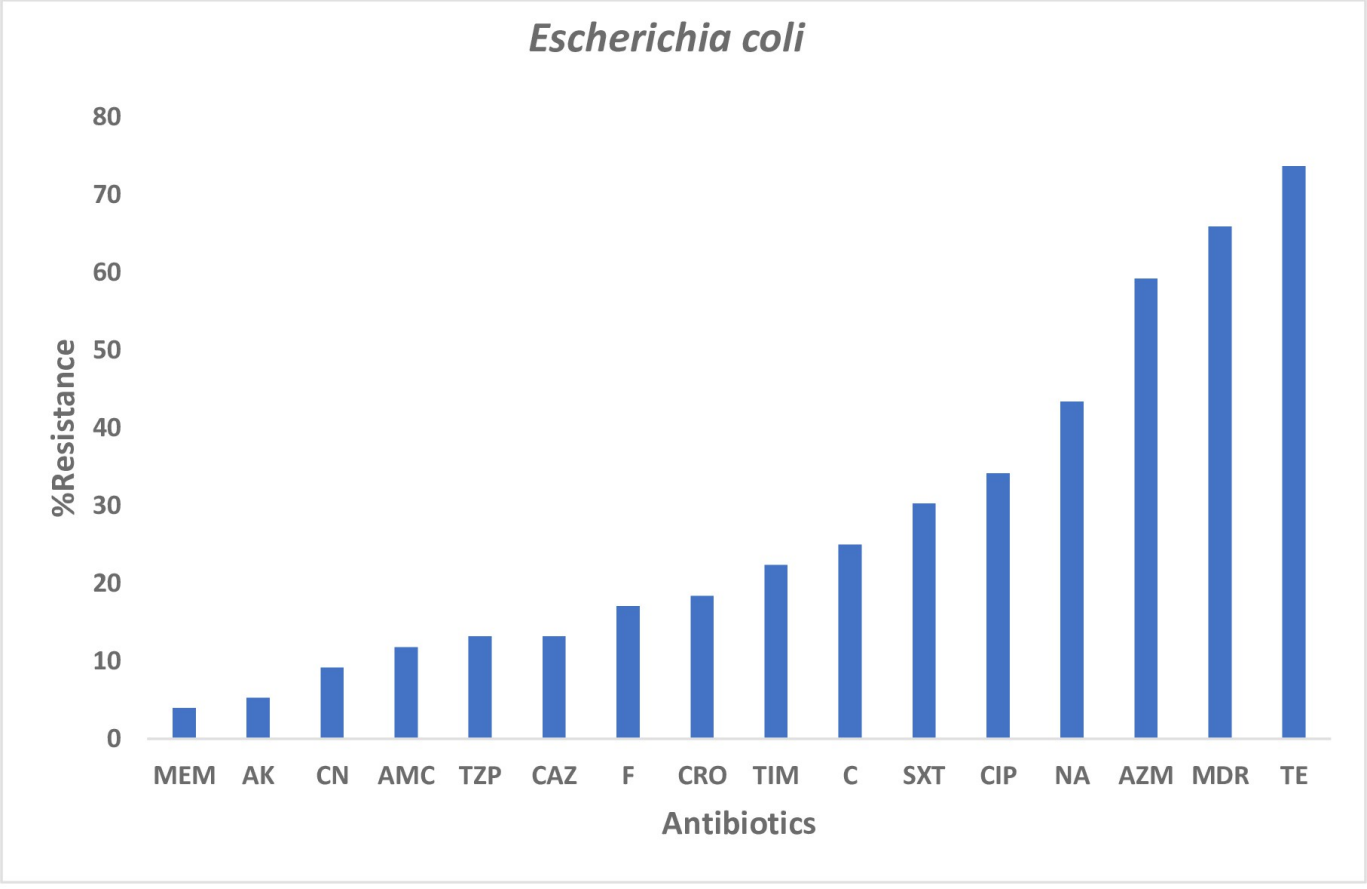
AMR profile among *E*. *coli* stool isolates. This shows the resistance profile of all the stool *E*. *coli* isolates recovered in the study.

Among the *E*. *coli* isolates that were recovered, tetracycline (73.7%) showed the highest resistance, 59.2% were resistant to Azithromycin, a macrolide (Fig 1). Non-susceptibility to fluoroquinolones (Nalidixic acid and Ciprofloxacin) and Sulfamethoxazole-trimethoprim was above 30%. Less resistance to Meropenem was observed (4%) among the *E*. *coli* isolates. Resistance to more than three classes of antibiotics (MDR) was detected in 65.9% of the isolates.

**Fig 2:**
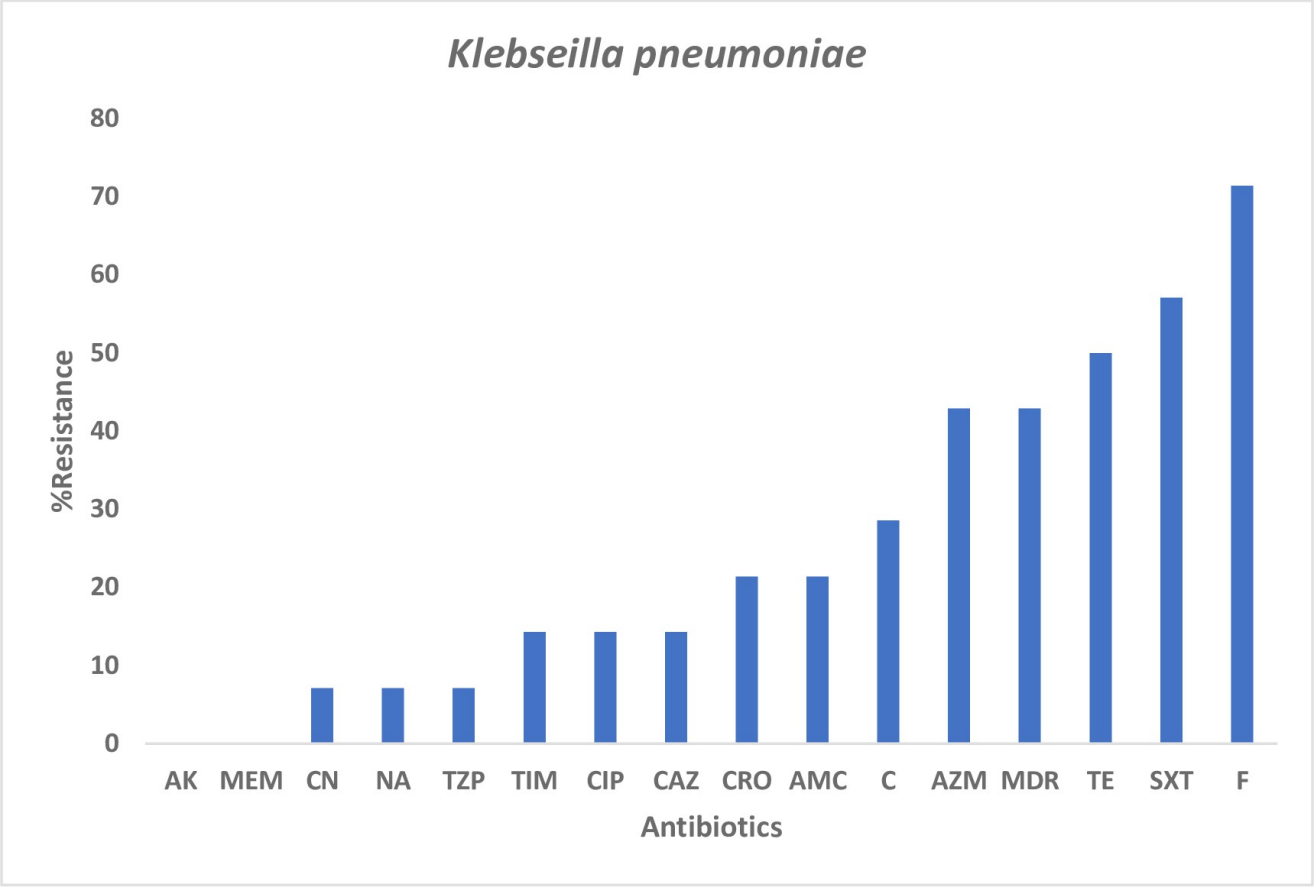
AMR profile among *K*. *pneumoniae* stool isolates. Here the figure shows various antibiotics that show resistance among the *K*. *pneumoniae* stool isolates.

Among the *K*. *pneumoniae* isolates, Nitrofurantoin (71.4%) showed the highest resistance, followed by Sulfamethoxazole-trimethoprim (57.1%) and Tetracycline (50%) with an MDR of 42.9% (Fig 2).

The *Citrobacter freundii* isolate was resistant to Nitrofurantoin, Sulfamethoxazole-trimethoprim, Chloramphenicol and Nalidixic acid.

Out of the 7 *Proteus mirabilis* that were recovered in the stool isolates, all isolates showed resistance to Nitrofurantoin and Gentamicin. A total of 86% were MDR and showed resistance to Tetracycline and Azithromycin.

However, patients with ESBL producing diarrhoea were more likely to be MDR’s than patients with non ESBL producing diarrhoea (89.5% vs. 62.5%; p = 0.027). ESBL samples had a significant association with resistance to multiple antibiotics (Sulfamethoxazole-trimethoprim, Ticarcillin-clavulanate, Nitrofurantoin, Gentamicin, Nalidixic acid, Piperacillin-tazobactam, Chloramphenicol, Ciprofloxacin) compared to those who did not have the ESBL properties (Table 3).

**Table 3:** Relationship between antibiotic resistance and ESBL/ non ESBL associated diarrhoea.

Characteristics	Total	ESBL Producing diarrhoea	Non ESBL Producing diarrhoea	*p-value*
		n(%)	n(%)	
**SXT**				
Resistant	58	17(89.5)	41(64.1)	0.046
Non-Resistant	25	2(10.5)	23(35.9)	
**TIM**				
Resistant	20	10(52.6)	10(15.6)	0.002
Non-Resistant	63	9(47.4)	54(84.4)	
**TE**				
Resistant	61	15(79.0)	46(71.9)	0.768
Non-Resistant	22	4(21.1)	18(28.1)	
**F**				
Resistant	29	12(63.2)	17(26.6)	0.006
Non-Resistant	54	7(63.2)	47(73.44)	
**CN**				
Resistant	8	5(26.3)	3(4.7)	0.014
Non-Resistant	75	14(73.7)	61(95.3)	
**NA**				
Resistant	37	12(63.2)	25(39.1)	0.073
Non-Resistant	46	7(36.8)	39(60.9)	
**TZP**				
Resistant	11	6(31.6)	5(7.8)	0.015
Non-Resistant	72	13(68.4)	59(92.2)	
**AZM**				
Resistant	55	14(73.7)	41(64.1)	0.583
Non-Resistant	28	5(26.3)	23(35.9)	
**AK**				
Resistant	4	1(5.3)	3(4.7)	1.000
Non-Resistant	79	18(94.7)	61(95.3)	
**C**				
Resistant	27	10(52.6)	17(26.6)	0.050
Non-Resistant	56	9(47.4)	47(73.4)	
**MEM**				
Resistant	4	1(5.3)	3(4.7)	1.000
Non-Resistant	79	18(94.7)	61(95.3)	
**CIP**				
Resistant	28	11(57.9)	17(26.6)	0.025
Non-Resistant	55	8(42.1)	47(73.4)	
**CRO**				
Resistant	18	14(73.7)	4(6.3)	<0.001
Non-Resistant	65	5(26.3)	60(93.8)	
**CAZ**				
Resistant	12	11(57.9)	1(1.6)	<0.001
Non-Resistant	71	8(42.1)	63(98.4)	
**AMC**				
Resistant	13	3(15.8)	10(15.6)	1.000
Non-Resistant	70	16(84.2)	54(84.4)	
**Multidrug Resistance**				
MDR	26	17(89.5)	40(62.5)	0.027
Not MDR	57	2(10.5)	24(37.5)	
**Total**	**83**	**19**	**64**	-

Key: SXT- Sulfamethoxazole-trimethoprim; TIM- Ticarcillin-clavulanate; TE-Tetracycline; F-Nitrofurantoin; CN-Gentamicin; NA- Nalidixic acid; TZP- Piperacillin-tazobactam; AZM- Azithromycin; AK-Amikacin; C- Chloramphenicol; MEM-Meropenem; CIP- Ciprofloxacin; AMC- Amoxicillin-clavulanate.

**Fig 3:**
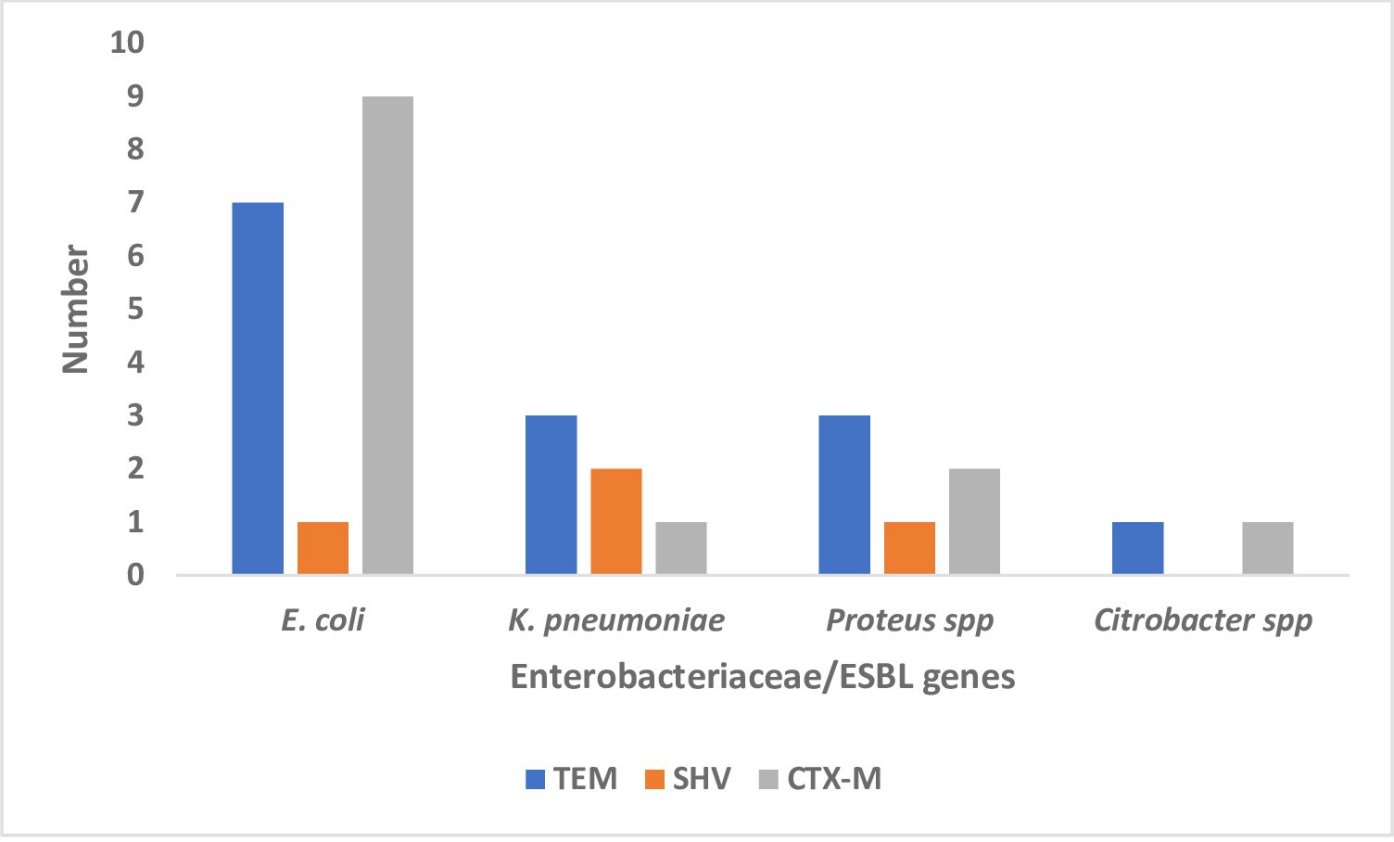
Specific ESBL genes tested among the various Enterobacteriaceae isolates. This shows the presence/ absence of *bla*_*CTX-M*_, *bla*_*SHV*_ and *bla*_*TEM*_ genes from phenotypically positive isolates among the *E*. *coli*, *K*. *pneumoniae* and *Proteus mirabilis*, and *Citrobacter freundii* isolates recovered in the stool samples.

Of all the ESBL genes that were recovered, TEM showed the highest occurrence as compared to the other genes (Fig 3) (S1 Raw image). The TEM, SHV and CTX-M genes were shown among some of the *E*. *coli*, *K*. *pneumoniae*, *Proteus mirabilis*, and *Citrobacter freundii* although the SHV gene was not identified in the *Citrobacter freundii* isolate (Fig 3).

### Diarrhoeagenic *E*. *coli*

None of the *E*. *coli* samples tested positive for DEC genes (*stx*1, *stx*2, *eae*, *stII*, virF, ipaH, *aafII*, *daaE*), except the Lt gene of ETEC which showed 34 positives (S2 Raw image). However, only 6 *E*. *coli* samples possessed both the ESBL genes and the Lt gene of ETEC as well. On the other hand, 4 *E*. *coli* isolates that were ESBLs were not ETEC.

## Discussion

ESBL and DEC detection play a very important role in understanding the levels of resistance and virulence in Enterobacteriaceae [17].

The purpose of this study was to show the presence of ESBL/DEC genes, as well as the AMR profile of the Enterobacteriaceae organisms recovered from the stool isolates.

Specific pathogens recovered in this study, showed an overall occurrence of ESBLs of 20.4% in *E*. *coli* (13.2%), *K*. *pneumoniae* (35.7%;), *Proteus mirabilis* (57.1%) and *Citrobacter freundii* (100%). This is similar to findings presented by Mahamat et al., 2019 where ESBL-PE fecal carriage among both hospitalized patients and healthy volunteers was 44% with *E*. *coli* showing the highest prevalence (72%), followed by *K*. *pneumoniae* (18%) and *Citrobacter freundii* (2%) [14]. This warrants extensive gastrointestinal laboratory testing for diarrhoeal patients due to the causative organism being a commensal/normal gut flora, yet having AMR properties that could either cause harm or be transferred to other pathogens to cause harm [30].

Also, ESBL among patients who had returned to Sweden with travelers’ diarrhoea, showed an overall prevalence of 24% (58/242), of which all pathogens were *E*. *coli* [3]. Various studies have shown *E*. *coli* to be one of the most common foodborne pathogens with ESBL properties indicating it’s tendency to spread in the community, therefore the need to track the transmission patterns to avoid foodborne epidemics in the future [25, 30] with increased morbidity and impact on DALYs. In Ghana, animal products to human transmission patterns have been investigated and have demonstrated to be a factor in the transmission of ESBL *E*. *coli* and its associated genes [18, 31, 32].

The presence of ESBL in *K*. *pneumoniae* is frequently concurrent with ESBLs in *E*. *coli* in most parts of Africa and Ghana [25, 33, 34]. Evidence from other ESBL related studies in Ghana have shown high prevalence of ESBL mainly among *E*. *coli*, *K*. *pneumoniae* and other organisms such as *Proteus* spp, *Citrobacter* spp and *Enterobacter* spp among blood, serum, stool, surgical site infections, sputum among others [15, 19, 22, 33–35]. Occurrence of the types of ESBL pathogens mentioned in this study is however consistent with the already existing and circulating pathogens that have been reported worldwide and a clear indication of diarrhoea surveillance as a means of identifying various types of AMR genes [25, 30, 32, 36–40].

Despite the low numbers of infants and children that were recruited during the study, 7 out of 23 isolates were ESBL causing diarrhoea as compared to 12 ESBLs from a total of 75 isolates of the adults. Children have shown high ESBL fecal carriage in several studies that have been conducted in Ghana and Nigeria [18, 33, 37, 40]. This could be attributed to a difference in health care seeking behaviour in relation to diarrhoea treatment in children and partly due to availability of over-the-counter medication/without prescription.

The high MDR occurrence observed in association with ESBL (89.5%) shows the effect bacteria with ESBL properties have with resistance to multiple drugs in Ghana. Although resistance to Tetracycline was high (79%) among the ESBL organisms recovered, there was no statistical significance between Tetracycline and ESBL related diarrhoeal infections. However, other enteric studies in Ghana have recorded over 50% resistance of Tetracycline [15, 41]. Co-resistance to Sulfamethoxazole-trimethoprim was high (89.5%) among ESBL causing diarrhoea showing how the drug has remained ineffective in the treatment of gastrointestinal infections in some parts of Ghana and Tanzania [39, 42]. Other antibiotics such as Chloramphenicol, beta lactams (Piperacillin-tazobactam, Ticarcillin-clavulanate), the fluoroquinolones (Ciprofloxacin, Nalidixic acid), the aminoglycoside, Gentamicin and Nitrofurantoin that showed a general highly ineffectiveness in the treatment of ESBL related diarrhoeal disease concurred with other previous Ghanaian studies that had been conducted [4, 22, 33, 41]. This is indicative of a gradual narrowing of the most routinely prescribed and readily available treatment options for diarrhoea in the country due to the selective pressure that may have been placed on these antibiotics over the years, as well as the over-the-counter availability of some these antibiotics as well [39]. Our findings in relation to MDR occurrence and the various classes of AMR was consistent with similar findings from Ethiopia [38], Tanzania [37, 39], Nigeria [40] and Chad [14]. Decreased resistance to meropenem, a major public health concern in Ghana was observed in the study. The gradual resistance shows possible abuse after its introduction on the Ghanaian market since 2002, therefore limiting treatment options [33].

Our findings showed that CTX-M gene had a high occurrence, consistent with a wide dissemination in Europe, Asia and Africa, which is attributed to ESBL activity by horizontal gene transfer aside other genetic factors [25, 32, 34]. Other Ghanaian studies had earlier indicated CTX-M gene to be the most frequent ESBL type occurring among ESBL-producing bacteria [18, 35, 43]. Diarrhoea-related ESBL studies in other countries have also reported CTX-M to be more frequent than the other ESBL types [30, 37, 40]. The TEM gene on the other hand also showed the highest occurrence in the study. It was solely detected in an earlier study conducted in Ghana [19], inconsistent with its common coexistence with CTX-M gene in many studies reported elsewhere [25, 36, 40]. Only few occurrence of the SHV gene (4/20) was recovered which was consistent with diarrhoeal studies carried out elsewhere despite the high concurrence of CTX-M and TEM genes [30, 37, 40, 44].

*Proteus* spp., normally ignored during microbiological analysis in diarrhoeagenic situations due to its commensal relevance in the gut showed high occurrence of ESBL phenotype and genotype as well as MDR properties in this study despite the few numbers recovered (4/7). Therefore, it should not be overlooked due to its hypermotility ability and biofilm domination leading to possible AMR transfer to other bacterial organisms [18].

The heat labile ETEC gene was the only DEC that occurred in this study. Although inconsistent with Opintan et al.’s study which recovered two other types of DEC genes (EAEC and EPEC), our results showed a significant association of DEC with diarrhoea among infants [7]. The findings from this study suggest, ETEC could be circulating and possibly endemic in the region. ETEC is well known to cause travelers’ and childhood diarrhoea leading to dehydration while its occurrence could also cause diarrhoea among adults living in endemic areas [6]. *E*. *coli* is gradually increasing in pathogenicity, virulence and association with AMR in humans, animals and the environment, it is therefore important to investigate its diarrhoeagenic properties for a better understanding of these mechanisms [4–8].

## Limitations

The present study was carried out under operational and material constraints due to the Covid-19 pandemic leading to reduced sample size and the inability to perform enhanced molecular tests as well as Whole Genome Sequencing (WGS) on some selected ESBLs as well as DEC. Despite the numerous genetic variants of ESBLs, only three enzymes (CTX-M, SHV and TEM) were investigated resulting in limited information of the possible variants that could be found among the ESBL-PE. Although ESBLs are mostly plasmid-mediated, due to limited resource, this study could not conduct any experiment to investigate this phenomenon.

## Conclusion

This study has confirmed the presence and antimicrobial resistance profile of ESBLs in most isolated bacteria in diarrhoeagenic stool samples. This shows the current resistance could exacerbate if more diarrhoea AMR research focused studies are not implemented. Routine ASTs during surveillance and hospital diagnostics should be encouraged to identify increasing and/ or upcoming AMR like carbapenem resistance among ESBLs, an upcoming public health concern which calls for better antibiotic stewardship. The observation of the Lt gene among *E*. *coli* isolates, a major virulent property among ETECs indicates the chances of increasing severity of diarrhoeal disease especially in children and the potential spread of AMR in gut bacteria. Frequent investigation of DEC should be encouraged to understand its epidemiology and clinical implications in Ghana better.

## Supporting information

S1 Raw image Fig 1 Multiplex PCR assay for detecting *bla*_*CTX-M*_, *bla*_*SHV*_ and *bla*_*TEM*_ genes from phenotypically positive isolates. (TIF)

S2 Raw image Fig 2 PCR assay for the detection of the Lt gene of Enterotoxigenic *E*. *coli* (ETEC) from *E*. *coli* isolates. (TIF)
