# Supplementary figures and images for: Diarrhoeagenic E. coli occurrence and antimicrobial resistance of Extended Spectrum Beta-Lactamases isolated from diarrhoea patients attending health facilities in Accra, Ghana
Source: PLoS One. 2022 May 26;17(5):e0268991. doi: 10.1371/journal.pone.0268991 (PMC9135277; doi:10.1371/journal.pone.0268991)

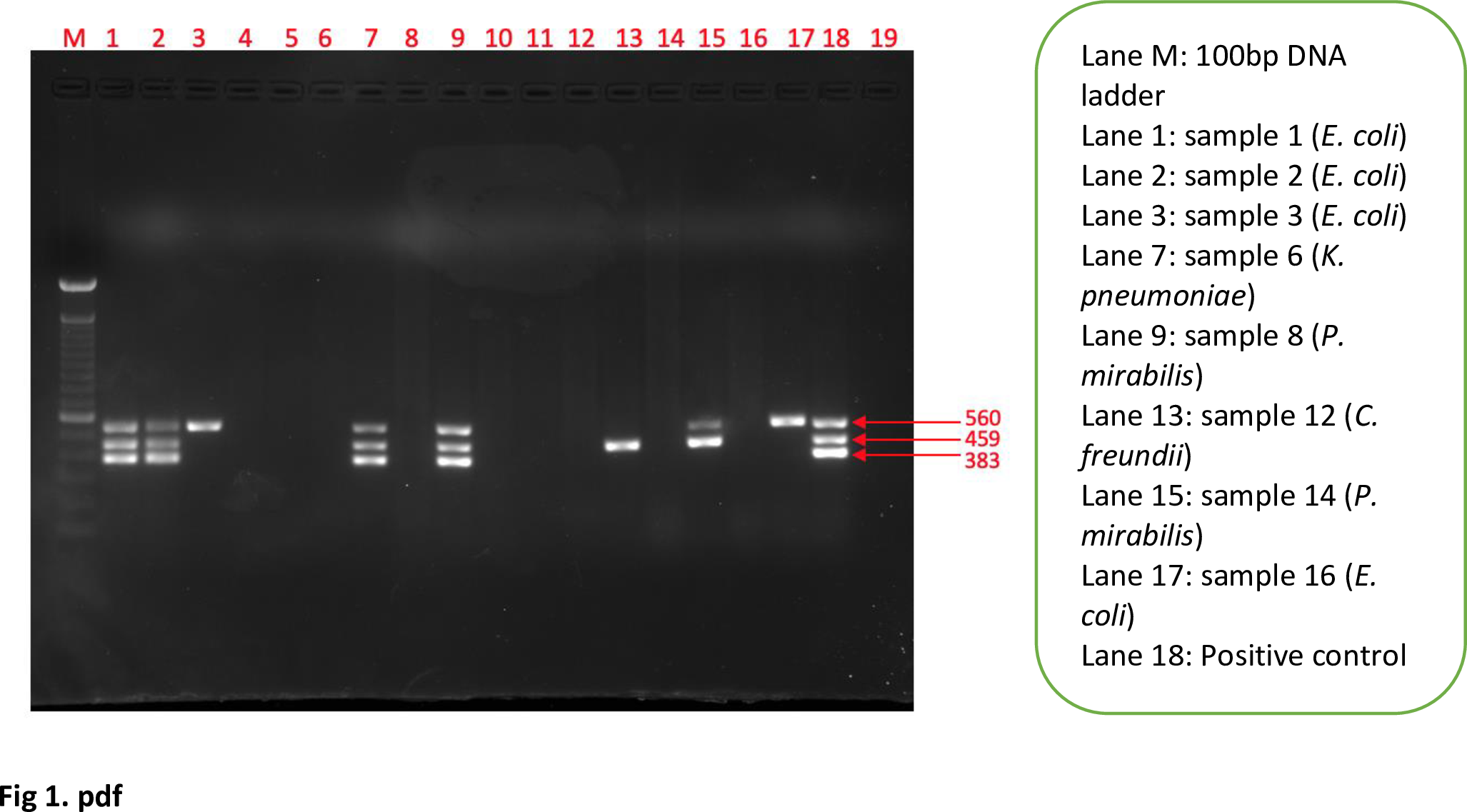

Supplement: S1 Raw image — (TIF) [file pone.0268991.s001.tif]

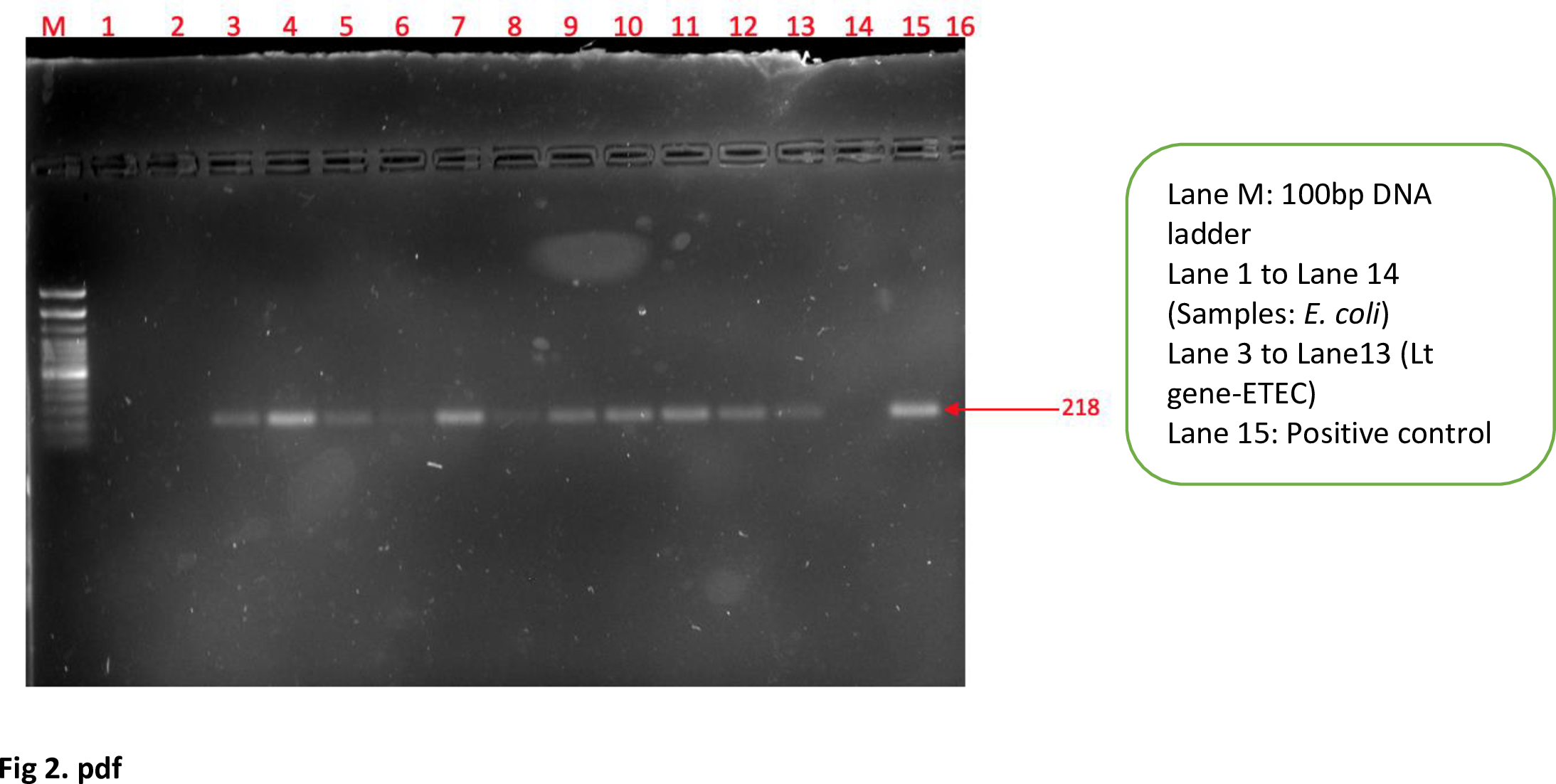

Supplement: S2 Raw image — (TIF) [file pone.0268991.s002.tif]
